# Optimization of the clinically approved mg-Zn alloy system through the addition of ca

**DOI:** 10.1186/s40824-022-00283-5

**Published:** 2022-09-05

**Authors:** Hyung-Jin Roh, Jaeho Park, Sun-Hee Lee, Do-Hyang Kim, Gwang-Chul Lee, Hojeong Jeon, Minseong Chae, Kang-Sik Lee, Jeong-Yun Sun, Dong-Ho Lee, Hyung-Seop Han, Yu-Chan Kim

**Affiliations:** 1 Nanostructural Material Laboratory, Department of Advanced Materials, Yonsei University, Seoul, 03722 Republic of Korea; 2 Center for Biomaterials, Korea Institute of Science and Technology, Seoul, 02792 Republic of Korea; 3 Research and Development Center, U&I Corporation, Uijongbu, 480-050 Republic of Korea; 4 Department of Materials science and Engineering, Seoul National University, Seoul, 08826 Republic of Korea; 5 KU-KIST Graduate School of Converging Science and Technology, Korea University, Seoul, 02841 Republic of Korea; 6 Biomedical Engineering Research Center, Asan Institute for Life Sciences, Asan Medical Center, College of Medicine, University of Ulsan, Seoul, 05505 Republic of Korea; 7 Department of Orthopedic Surgery, Asan Medical Center, College of Medicine, University of Ulsan, Seoul, 05505 Republic of Korea

**Keywords:** Biodegradable metal, Micro-galvanic corrosion, Mechanical properties, Corrosion resistance, In vitro, Orthopedic implant, Mg alloy

## Abstract

**Background:**

Although several studies on the Mg-Zn-Ca system have focused on alloy compositions that are restricted to solid solutions, the influence of the solid solution component of Ca on Mg-Zn alloys is unknown. Therefore, to broaden its utility in orthopedic applications, studies on the influence of the addition of Ca on the microstructural, mechanical, and corrosion properties of Mg-Zn alloys should be conducted. In this study, an in-depth investigation of the effect of Ca on the mechanical and bio-corrosion characteristics of the Mg-Zn alloy was performed for the optimization of a clinically approved Mg alloy system comprising Ca and Zn.

**Methods:**

The Mg alloy was fabricated by gravitational melting of high purity Mg, Ca, and Zn metal grains under an Ar gas environment. The surface and cross-section were observed using scanning electron microscopy (SEM) and transmission electron microscopy (TEM) to analyze their crystallographic structures. Electrochemical and immersion tests in Hank’s balanced salt solution were used to analyze their corrosion resistance. Tensile testing was performed with universal testing equipment to investigate the impact of Ca addition. The examination of cytotoxicity for biometric determination was in line with the ISO10993 standard.

**Results:**

In this study, the 0.1% Ca alloy had significantly retarded grain growth due to the formation of the tiny and well-dispersed Ca_2_Mg_6_Zn_3_ phase. In addition, the yield strength and elongation of the 0.1% Ca alloy were more than 50% greater than the 2% Zn alloy. The limited cell viability of the 0.3% Ca alloy could be attributed to its high corrosion rate, whereas the 0.1% Ca alloy demonstrated cell viability of greater than 80% during the entire experimental period.

**Conclusion:**

The effect of the addition of Ca on the microstructure, mechanical, and corrosion characteristics of Mg-Zn alloys was analyzed in this work. The findings imply that the Mg-Zn alloy system could be optimized by adding a small amount of Ca, improving mechanical properties while maintaining corrosion rate, thus opening the door to a wide range of applications in orthopedic surgery.

## Introduction

Recent advancement in the field of biodegradable metals has led to the development and clinical study of the next generation of implantable devices that reduce the need for secondary surgery [1]. As a metallic implant material, Mg has biodegradability, biocompatibility, and a similar Young’s modulus to the bone, which alleviates the stress-shielding effect [2]. However, the application of pure Mg (P-Mg) is limited in load-bearing orthopedic implants due to its poor mechanical properties and relatively high corrosion rate depending on impurities [1]. Therefore, several alloy systems have been developed to overcome these shortcomings. Alloying elements, such as Al, Li, Zr, and rare earth metals, were initially considered due to their beneficial effect on mechanical properties [3]. Although these alloy systems demonstrated good biocompatibility in vitro and in vivo experiments, the possible side effects of rare earth metals and aluminum on human organs remain debatable [4–6].

Cha et al. studied the Mg alloy system consisting of Ca and Zn in an attempt to eliminate any possible side effects [7]. Ca and Zn were chosen as alloying elements due to their biocompatibility and grain refinement effect on Mg [8]. Ca is one of the main elements in human bone [9, 10], while Zn strengthens immunity and other biological mechanisms in the body [9, 11, 12]. Successful clinical application of the Mg alloy system with Ca and Zn was reported for small hand and wrist fractures with no detrimental effects after 1 year [13]. A recent study also showed that the Mg-Zn-Ca system exhibits a highly improved formability compared to the commercially available Mg-Al alloy [14]. However, the main drawback of the alloy system consisting of Zn and Ca is a high degradation rate induced by micro-galvanic corrosion [15–18]. Solution treatment can improve corrosion resistance by reducing the secondary phase; however, it decreases the mechanical strength [1, 19]. There have been studies to improve both mechanical and corrosion properties through the fine distribution of secondary phases by hot-working methods (e.g., extrusion and rolling). However, despite the improved mechanical properties of the Mg-Zn-Ca alloy, the corrosion rate is still greater than in extremely pure (99.9%) P-Mg [20–22].

Controlling a small range of alloying elements could be key to improving corrosion resistance in the Mg-Zn-Ca alloy. According to previous studies, unsolved secondary phases during heat-treatment also exist after plastic deformation, which can retard the uniform distribution of the secondary phase. The maximum solubility of Ca in Mg is only 0.2 wt% at 23 °C and 1.35 wt% at 516.5 °C in the equilibrium state. When 4 wt% Zn is added, the maximum solubility of Ca in Mg further reduces to 0.04 wt% at 368 °C [23]. Therefore, a small amount of Ca can greatly influence the properties of the Mg-Zn-Ca system due to significant changes in the distribution of secondary phases [23]. However, many studies on the Mg-Zn-Ca system have focused on alloy compositions that are limited to solid solutions, but have not been able to clearly understand the effect of the solid solution component of Ca on Mg-Zn alloys [20, 21, 24, 25].

Here, an in-depth evaluation of the influence of Ca on the mechanical and bio-corrosion properties of Mg-Zn alloy was performed for further optimization of a clinically approved Mg alloy system consisting of Ca and Zn. The small range of 0, 0.1, and 0.3 wt% Ca was studied to evaluate the differences between the Mg-2% Zn-0.1% Ca alloy with a solid solution region at high temperature and Mg-2% Zn-0.3% Ca alloy without a solid solution region.

## Materials and methods

### Sample preparation

The Mg alloy was prepared using the gravitational melting method, using high purity Mg (99.99%), Ca (99.5%), and Zn (99.9%) metal grains under an Ar gas atmosphere. The Mg alloy was prepared at a temperature of 740 °C for 100 min. The molten metal was then poured into a mold preheated at 200 °C. The as-cast alloy was heat-treated at 420 °C for 20 h. This was followed by quenching in water. The alloy was then extruded at 320 °C, with an extrusion ratio of 39.25 and ram speed of 0.2 mm/s. Table 1 shows the inductively coupled plasma results of the alloys used in this study.

**Table 1:** Chemical composition of the as-extruded Mg-alloys

Materials	Element (unit: wt%)						
	Ca	Zn	Fe	Ni	Mn	Si	B
2%Zn	–	1.90	< 0.003	< 0.002	0.0011	< 0.001	< 0.002
0.1%Ca	0.10	2.00	< 0.002	< 0.002	< 0.001	< 0.001	< 0.002
0.3%Ca	0.28	2.03	< 0.003	< 0.002	0.0011	< 0.001	< 0.002

### Microstructure

The microstructure of the alloy was observed during sample preparation with scanning electron microscopy (SEM, inspect F50). The alloy was observed at the following stages of its preparation: (i) the as-cast state, (ii) the as-heat treated state, (iii) the as-extruded state, and (iv) after immersion in Hank’s solution. For microstructure observation, samples at each stage of preparation were cut, ground with SiC papers (up to 2000 grit), and finely polished with diamond powders (0.25–3 μm). Energy dispersive spectroscopy (EDS) was used to characterize the chemical compositions of the alloy phases. The selected area diffraction pattern (SADP) of precipitates was investigated using transmission electron microscopy (TEM, TitanTM80–300).

### Immersion tests

The immersion tests were carried out in Hank’s balanced salt solution. The pH and temperature of Hank’s solution were adjusted to 7.4 and 37 °C, respectively. Coin samples with a diameter of 8 mm and thickness of 1 mm were prepared from the extruded alloy. They were ground with SiC paper (up to 2000 grit), ultrasonically cleaned in ethanol for 10 min, dried in air, and immersed in Hank’s solution. The hydrogen gas generated was collected using a funnel system to measure the corrosion rate. Considering the area and immersion time of the samples, the H_2_ generation rate was calculated and represented as V_H_ (ml/(cm^2^ day). The corrosion rate R_H_ (mm/y) is related to the H_2_ generation rate V_H_ (ml/(cm^2^ day), according to Eq. (1).

1 $${R}_H=2.279\ {V}_H$$

### Electrochemical measurements

The electrochemical measurements were conducted in a three-electrode cell using a potentiostat (EG&G PAR Model 263A Potentiostat/Galvanostat). The Mg alloy was used as the working electrode. Platinum and Ag/AgCl were used as counter and reference electrodes, respectively. A potential range was applied to the working electrode at a scanning rate of 0.83 mV/s. The corrosion current density (i_corr_) was estimated using linear regression and Tafel extrapolation to the cathodic and anodic sections of the polarization curves. The corrosive environment had a pH of 7.4. Hank’s solution was left in air at 36.5 °C. The area of the samples exposed to the electrolyte was 0.28 cm^2^. The corrosion rate was calculated with Eq. (2).

2 $$\mathrm{Corrosion}\ \mathrm{rate}\ \left(\frac{\mathrm{mm}}{\mathrm{yr}}\right)=3.28\times \frac{\mathrm{MI}}{\mathrm{nd}}$$

M: Atomic mass (u).

I: Corrosion current density (mA cm^− 2^).

n: Number of electrons involved in the corrosion reaction.

d: Density (g cm^− 3^).

The open circuit potential (OCP) of the intermetallic compound was measured for 30 min. The sample preparation was the same as the fabrication of Mg alloys described above, and Ca_2_Mg_6_Zn_3_, Mg, and Mg_2_Ca samples with Zn were fabricated from custom-made ingots from R&D Korea.

### Mechanical properties

Tensile testing was conducted using a universal testing machine (MTS U.S.A). Samples for the tensile test were prepared according to ASTM B557–10. The speed of crosshead and gauge length at room temperature was 2 mm/min and 15 mm, respectively. To evaluate the change of mechanical properties after immersion, samples were immersed in Hank’s solution for 30 days. During this test, Hank’s solution was replaced every 4 days. After immersion, the corrosion products were removed with chromic acid (180 g/L CrO_3_ + 1 g/L AgNO_3_ in distilled water) for 1 min.

### Cytotoxicity test

Cytotoxicity evaluation for biometric determination is an example of progress in accordance with ISO10993 standards. The experimental method is as follows. After eluting the specimen in DMEM (Dulbecco’s Modified Eagles Medium), a cell culture solution, for 1, 3, and 7 days, vascular cells (L-929) were cultured in each solution for 1 day, and the number of surviving cells was determined. The number of surviving cells was determined by measuring the formazan absorbance using the CCK-8 kit with 1X DMEM containing no serum, and the ratio of the label to the solution was 1cm^2^/ml. The test was performed in an incubator maintaining a 37.5% CO_2_ atmosphere.

### Statistical analysis

One-way ANOVA with post hoc Tukey’s honest significant difference (HSD) test was used to examine the experimental groups. A statistically significant difference between two groups with *p* < 0.05, *p* < 0.01, and *p* < 0.001 is denoted by *, **, and ***, respectively.

## Results

### Microstructure

**Fig. 1:**
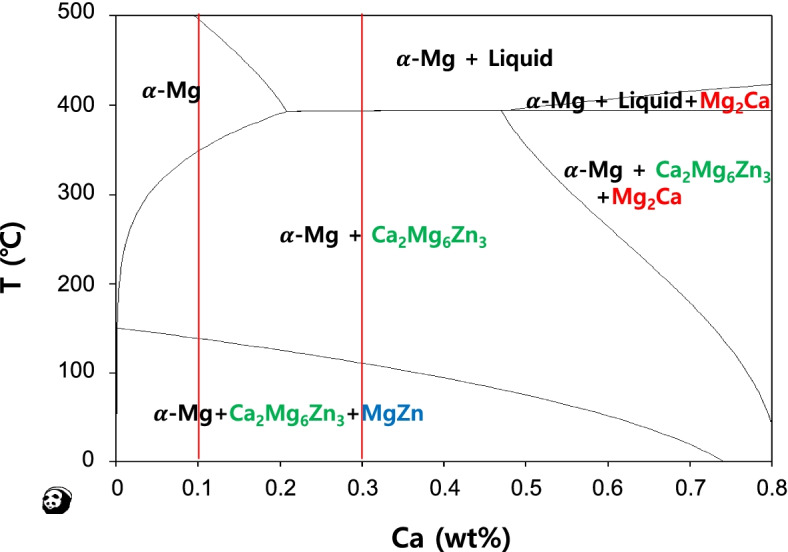
Dove phase diagram of Mg-2%Zn-Ca

**Fig. 2:**
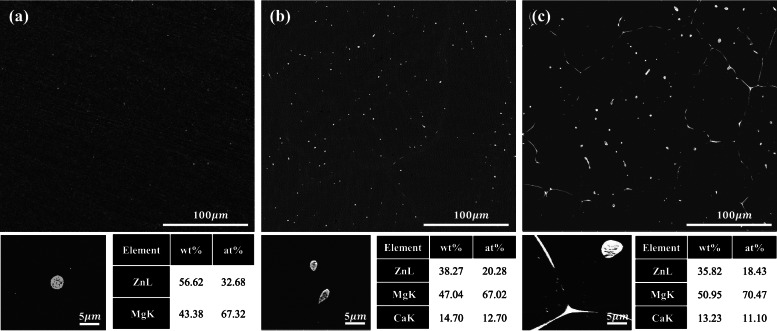
Microstructures of the as-cast alloys: (**a**) 2%Zn, (**b**) 0.1%Ca, and (**c**): 0.3%Ca

The addition of a small amount of Ca in the Mg-2% Zn alloy system was investigated to characterize the difference between the Mg-2% Zn-0.1% Ca alloy with a solid solution and Mg-2% Zn-0.3% Ca alloy without a solid solution region, as shown in the phase diagram (Fig. 1). The SEM images of as-cast Mg-2% Zn-xCa (x = 0, 0.1, 0.3 wt%) are shown in Fig. 2. The 2% Zn alloy showed the secondary phase with equiaxed grains (of size 3 μm), as displayed in Fig. 2a. As Ca was added to the 2% Zn alloy, the fraction of the secondary phase distributed along the grain boundary increased, as shown in Fig. 2b-c. EDS results and the phase diagram of Mg-Zn-Ca showed that the Mg-Zn phase was formed in the 2% Zn alloy, and the Ca_2_Mg_6_Zn_3_ phase was formed in the Ca added alloys. This is in good agreement with the previous studies, which reported that the addition of Ca in Mg-Zn alloys retards the formation of the Mg-Zn phase and promotes the formation of the Ca_2_Mg_6_Zn_3_ phase [26, 27].

**Fig. 3:**
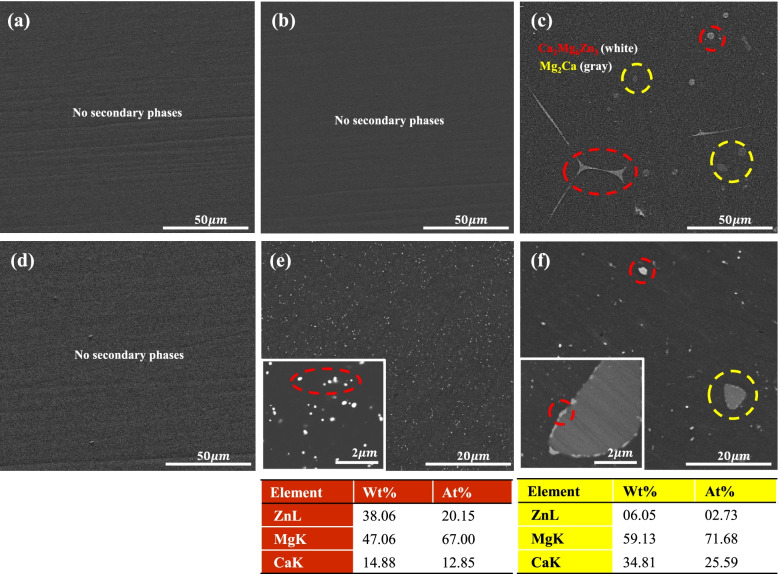
Microstructures of as-heat treated (**a**) 2% Zn, (**b**) 0.1% Ca, and (**c**) 0.3% Ca and as-extruded (**d**) 2% Zn (**e**) 0.1% Ca, and (**f**) 0.3% Ca alloys

Figure 3a–c show the SEM images of as-heat treated the 2% Zn alloy, 0.1% Ca, and 0.3% Ca alloys, respectively. After heat-treatment, secondary phases were not observed in the 2% Zn and 0.1% Ca alloys. This can be attributed to the dissolution of the secondary phase in the Mg matrix. Most of the Ca_2_Mg_6_Zn_3_ of the as-cast alloys dissolved in the Mg matrix. However, the equiaxed secondary phase was still observed in the 0.3% Ca alloy. According to EDS analysis, the white area (circled in red in Fig. 3) is Ca_2_Mg_6_Zn_3_ and the gray area (circled in yellow in Fig. 3) is Mg_2_Ca, which included small amounts of Zn (2.7 at%). The formation of the Mg_2_Ca phase has not been reported in other similar studies [7]. Figure 3d–f show the microstructure of as-extruded 2% Zn, 0.1% Ca, and 0.3% Ca alloys, respectively. The extruded alloy without Ca had no secondary phase, but the 0.1% Ca alloy (Fig. 3e) had fine (~ 200 nm) and well-dispersed Mg_6_Ca_2_Zn_3_. However, coarse (~ 4 μm) Mg_2_Ca, and small and well-dispersed Ca_2_Mg_6_Zn_3_ were observed in the 0.3% Ca alloy (Fig. 3f). The Ca content is also an important factor in the grain size of as-extruded alloys (Table 2). For the addition of small amounts of Ca in Mg-Zn alloys, increasing Ca content effectively decreases the grain size.

**Table 2:** Grain size of the as-extruded Mg-Zn-Ca alloys

Materials	2%Zn	0.1%Ca	0.3%Ca
Grain Size	24.83 ± 8.29 μm	6.45 ± 1.79 μm	1.12 ± 1.39 μm

**Fig. 4:**
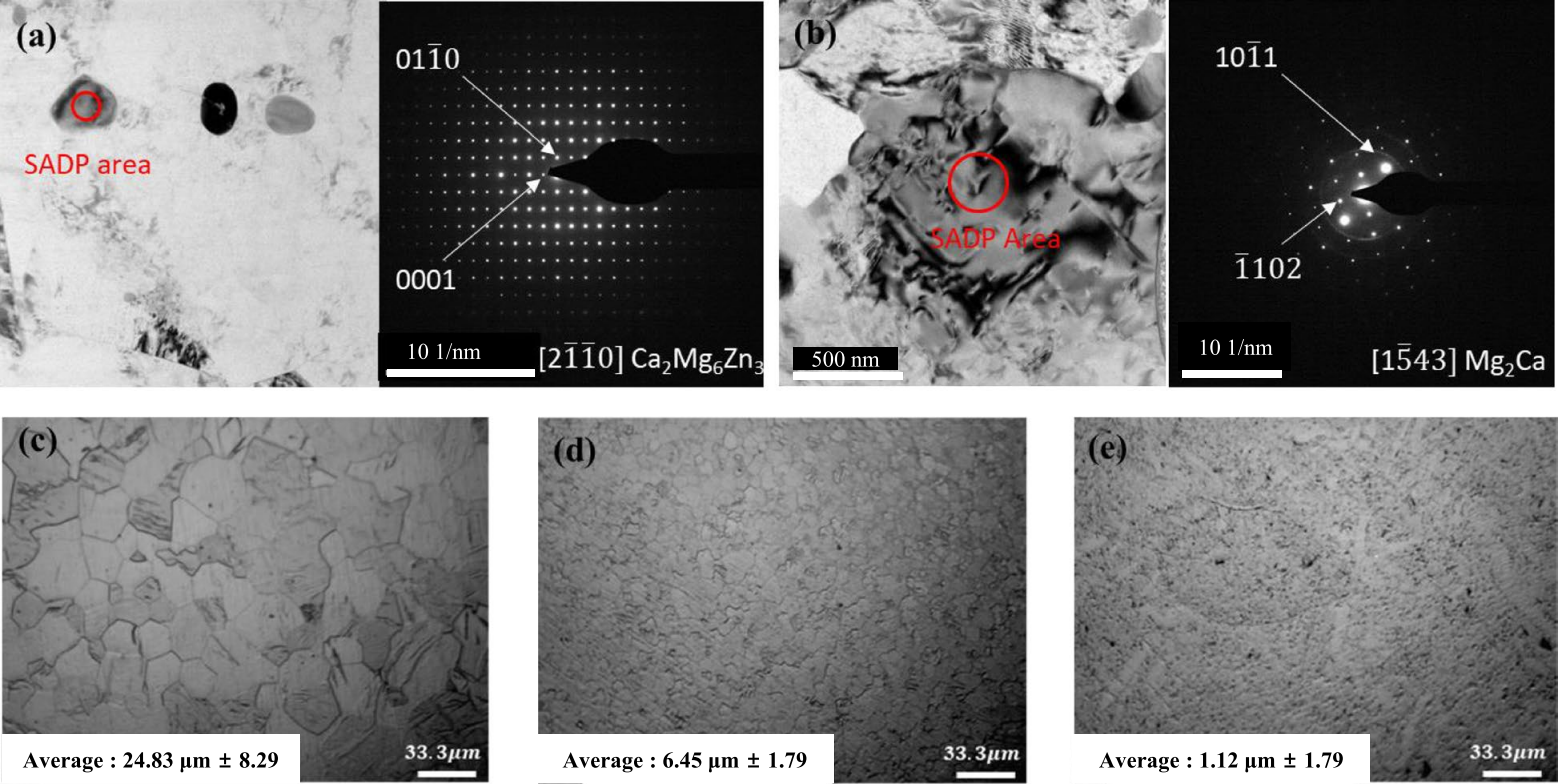
TEM bright field images of as-extruded (**a**) 0.1% Ca and (**b**) 0.3% Ca alloys and OM image of the extruded surface of (**c**) 2% Zn, (**d**) 0.1% Ca, and (**e**) 0.3% Ca alloys

The secondary phases in the extruded 0.1 and 0.3% Ca alloy were further analyzed by TEM. TEM bright-field images of extruded 0.1 and 0.3% Ca alloy are shown in Figs. 4a–b. Fig. 4a shows the bright field image of 0.1% Ca alloy that was taken with the incident beam along the [2 $\overline{1}\overline{1}$ 0] zone axis of the Mg matrix. The SADP of the secondary phase confirmed that it indeed was Ca_2_Mg_6_Zn_3_. Fig. 4b shows the bright field image of 0.3% Ca alloy that was taken with the incident beam along the [1 $\overline{5}$ 43] zone axis of the Mg matrix. The SADP of the secondary phase confirmed that these phases were Mg_2_Ca. Figure 4c–e show that the addition of small amounts of Ca in Mg-Zn alloys can effectively decrease the grain size from 24.83 ± 8.29 μm in 2% Zn to 6.45 ± 1.79 μm and 1.12 ± 1.39 μm in 0.1 and 0.3% Ca alloys, respectively.

### Corrosion properties

**Fig. 5:**
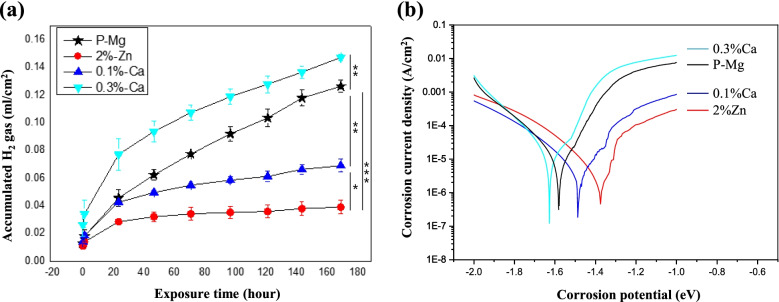
Results of the (**a**) immersion and (**b**) electrochemical corrosion tests

Figure 5 shows the results of immersion and electrochemical testing. Immersion testing was performed by collecting hydrogen gas from extruded samples immersed in Hank’s solution for 7 days. Generally, the degradation process of Mg in electrolytes can be expressed by the reaction in Eq. (3).

3 $$\mathrm{Mg}+2{\mathrm{H}}_2\mathrm{O}\longrightarrow \mathrm{Mg}{\left(\mathrm{OH}\right)}_2+{\mathrm{H}}_2$$

The 2% Zn alloy showed the lowest amount of hydrogen gas (0.038 ml/cm^2^) compared to the other samples. The hydrogen gas released for the other samples were 0.07, 0.123, and 0.148 ml/cm^2^ for 0.1% Ca alloy, P-Mg, and 0.3% Ca alloy, respectively. Figure 5b shows the polarization curves of the investigated samples. Generally, the cathodic polarization curve is related to the evolution of H_2_ gas through the de-oxygenation reaction of water. Meanwhile, the anodic polarization curve is related to the degradation of Mg and the formation of Mg^2+^ ions [28]. The corrosion potential and corrosion current density were calculated from the polarization curve by the Tafel extrapolation method. Results indicate that the corrosion potential of the investigated alloys decreased depending on the amount of added Ca. The gradients of the cathodic curve for the 0.3% Ca and P-Mg alloys were steeper than those of the other samples. However, the cathodic curve gradients for 2% Zn and 0.1% Ca alloys were relatively low in the applied potential range. The corrosion current density decreased to 17 × 10^− 6^, 14 × 10^− 6^, 4.9 × 10^− 6^ and 4.4 × 10^− 6^ A/cm^2^ for 0.3% Ca, P-Mg, 0.1% Ca, and 2% Zn alloys, respectively. The general trends of corrosion rate from the immersion and electrochemical tests were in good agreement. Calculated parameters from the electrochemical and immersion tests (corrosion potential, corrosion current density, and corrosion rate) are listed in Table 3.

**Table 3:** Electrochemical parameters and corrosion rates obtained by polarization and immersion testing

Materials	Corrosion potential (V)	Corrosion density (***μ*** A/cm^2^)	Polarization (mm/y)	Immersion (mm/y)
P-Mg	− 1.59	14	0.32	0.103
2%Zn	−1.44	4.4	0.10	0.064
0.1%Ca	−1.49	4.9	0.11	0.095
0.3%Ca	−1.62	17	0.39	0.172

**Fig. 6:**
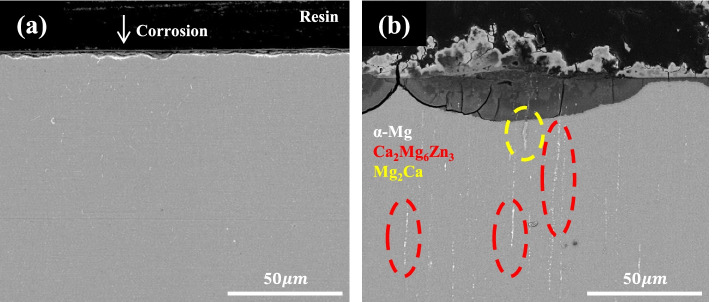
Cross-section view of the immersed samples of (**a**) 0.1% Ca and (**b**) 0.3% Ca

**Fig. 7:**
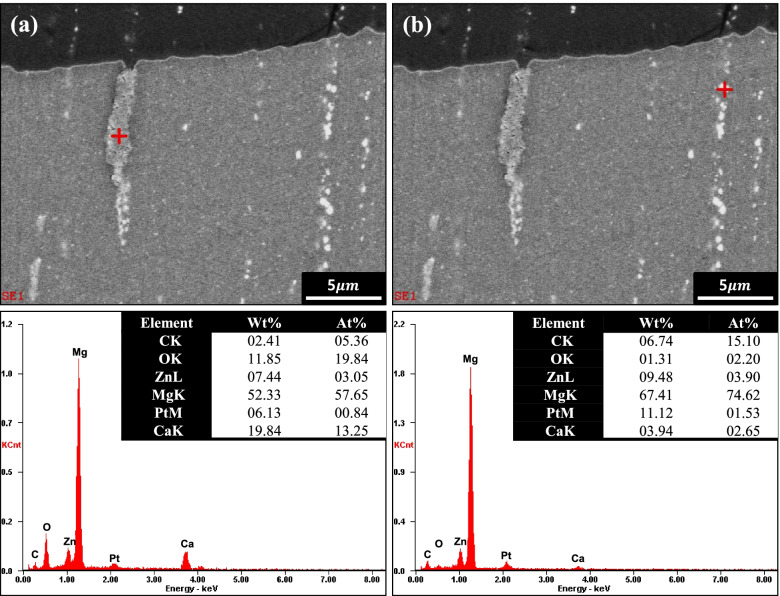
EDS results of the 0.3% Ca alloy for (**a**) Mg2Ca and (**b**) Ca_2_Mg_6_Zn_3_

**Fig. 8:**
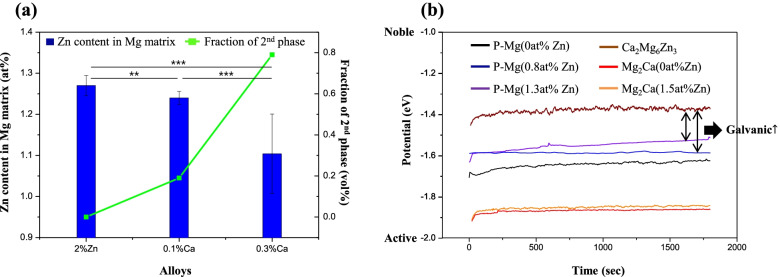
**a** Zn content in the Mg matrix of each alloy and (**b**) open circuit potential of phases

Figure 6 shows the microstructures of the cross-section of samples (0.1 and 0.3% Ca alloys) after immersion in Hank’s solution for 7 days. It was observed that the corrosion layer of the 0.1% Ca alloy (Fig. 6a) was uniform and very thin (~ 3 μm), while that of the 0.3% Ca alloy was thick (3–30 μm), and many signs of corrosion were detected. Corrosion in the vicinity of the coarse secondary phase along the extrusion direction seemed to be accelerated. Figure 7 shows the EDS results on the corroded surface of the 0.3% Ca alloy. Interestingly, local corrosion was found in the Ca-rich region (Mg_2_Ca), while local corrosion of Ca_2_Mg_6_Zn_3_ was not observed. Figure 8 shows the dependence of the fraction of the secondary phase and Zn content in the Mg matrix on the Ca content. There seems to be a relationship between the decrease in Zn content in the Mg matrix and the increase in the formation of the secondary phase. The secondary phase of the alloy, as calculated by the PANDAT program (ver. 7), increased with Ca content. This increase was notably higher from the 0.1–0.3% Ca alloy, when the Zn content in the Mg matrix decreased highly significantly. Figure 8b shows the open circuit potential (OCP) of the intermetallic compound. The potential value of the Ca_2_Mg_6_Zn_3_ was measured at − 1.38 V. The potential value of P-Mg increased with the addition of Zn, from − 1.68 V (0 at% Zn) to − 1.5 V (1.3 at% Zn). The potential of Mg_2_Ca also increased with the addition of Zn from − 1.9 V (0 at% Zn) to − 1.85 V (1.5 at% Zn).

In this experiment, the corrosion rate of the 0.1% Ca alloy was lower than that of P-Mg, which was similar to the 2% Zn alloy. However, the corrosion rate of 0.3% Ca alloy was higher than that of P-Mg. The dramatic variation of the corrosion rate within a small range of Ca content may be due to the behavior of Zn in the matrix. The addition of Zn improves corrosion resistance by increasing the potential of the Mg matrix [26]. However, the solubility of Zn in the Mg matrix decreased with the addition of Ca, leading to a gradual decrease in the Mg potential. A previous study demonstrated that corrosion resistance was achieved by controlling the potential gap of phases, even in alloys with many secondary phases [7]. Therefore, the corrosion behavior of Mg is affected by the potential differences between the secondary phases more than the volume fraction of the secondary phase.

In addition, the Mg_2_Ca phase (Fig. 8a) formed from heat treatment could accelerate local galvanic corrosion due to a lower potential than the Mg matrix. It has been reported that the formation of Mg_2_Ca can accelerate corrosion behavior by acting as an anodic electrode [8].

It was suggested by Hofstetter et al. that the degradation rate of the 5Zn-0.3Ca (wt%) is much higher than pure-Mg because Ca_2_Mg_6_Zn_3_ has a higher potential than the Mg matrix. However, in this study, it was found that the potential gap between Ca_2_Mg_6_Zn_3_ and the Mg matrix could be altered by controlling the amount of the alloy elements. The 0.1% Ca alloy showed excellent corrosion resistance.

### Mechanical properties

**Fig. 9:**
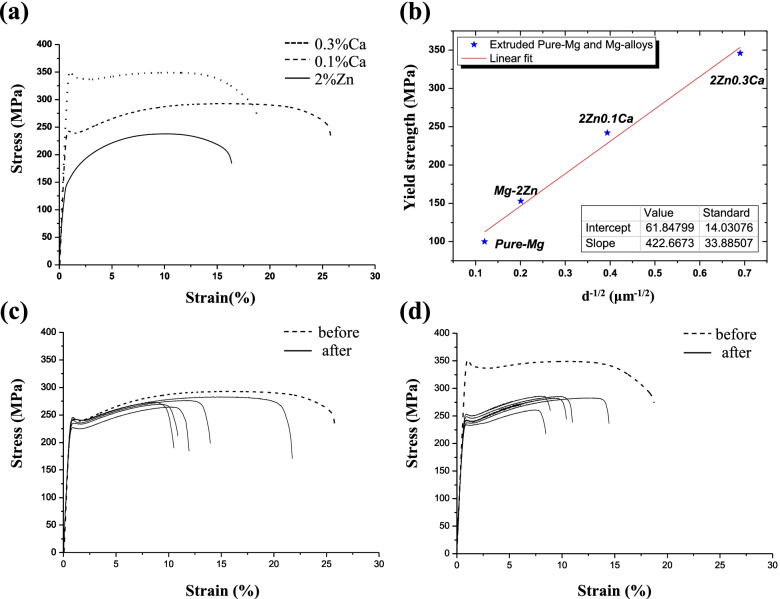
**a** Tensile test results of the investigated alloys. **b** relationship between yield strength and grain size. Tensile test results after immersion in Hank’s solution for 30 days of (**c**) 0.1% Ca and (**d**) 0.3% Ca alloys

Figure 9a shows the nominal stress-strain curve from the tensile test at room temperature. The yield strength, ultimate strength, and elongation to fracture of the 2% Zn alloy were 153.4 ± 1.2 MPa, 238.3 ± 0.8 MPa, and 16.2 ± 0.8%, respectively. Both strength and elongation increased for the 0.1% Ca alloy to 278.8 ± 5.2 MPa and 26.1 ± 1.6%. However, when the Ca content increased to 0.3%, the elongation of the alloy dropped from 26.1 to 17.4%, despite the dramatically increased strength. Figure 9b shows the Hall-Petch relationship between grain size and yield strength. With the addition of Ca, yield strength increases proportionally to the decrease in grain size. To evaluate the integrity of the mechanical properties in a corrosive environment, tensile testing on the samples with added Ca was conducted after the immersion test in Hank’s solution for 30 days. Figure 9c–d show the nominal stress-strain curve after immersion testing for 30 days in Hank’s solution. After the immersion test, the yield, ultimate strength, and elongation of the 0.1% Ca alloy were 239.4 ± 3.6 MPa, 276.3 ± 5.1 MPa, and 14.3 ± 4.0%, respectively. The strength of the 0.1% Ca alloy was maintained after immersion, while the elongation slightly decreased (Fig. 9c). However, the 0.3% Ca alloy showed a large reduction in strength, elongation, and deviation (Fig. 9d).

Many studies have demonstrated that the addition of Ca in Mg-Zn alloys can have a positive effect on mechanical properties [7]. The addition of Ca helps alloys generate a second phase during plastic deformation (e.g., during hot extrusion), which retards grain growth and causes the alloy to have a weak texture [8, 29]. This phenomenon occurs in the 0.3 wt% Ca alloy used in this study. Calculated parameters from the tensile test (yield strength, maximum strength, and elongation) are listed in Table 4.

**Table 4:** Tensile test results of the as-extruded Mg-Zn-Ca alloys

Materials	Yield strength (MPa)	Maximum Strength (MPa)	Elongation (%)
2%Zn	153.4 ± 1.2	238.3 ± 0.8	16.2 ± 0.8
0.1%Ca	242.4 ± 4.5	278.8 ± 5.2	26.1 ± 1.6
0.3%Ca	346.2 ± 4.5	338.9 ± 5.6	17.4 ± 0.5
0.1%Ca immersed	239.4 ± 3.6	276.4 ± 5.1	14.3 ± 4.0
0.3%Ca immersed	244.4 ± 10.6	276.9 ±9.6	10.1 ± 2.1

### Integrity: mechanical properties after immersion

**Fig. 10:**
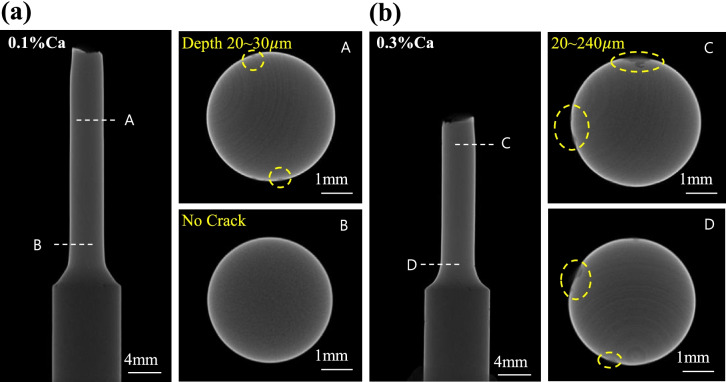
Observations of the corroded depth by using *μ*-CT of (**a**) 0.1% Ca and (**b**) 0.3% Ca alloys

Mg implants should be maintained for a certain period until suitable formation of new bone is achieved. Therefore, the strength after degradation in electrolytes is an important qualifying factor for an implant material. Tensile testing was conducted after immersion in Hank’s solution for 30 days. Interestingly, the mechanical strength of the 0.1% Ca alloy was maintained after immersion, while the mechanical strength of the 0.3% Ca alloy decreased drastically. Sun et al. reported that the mechanical strength of the 4wt% Zn-0.2wt% Ca alloy decreased to 70% after immersion for 30 days [20]. Both alloys (0.3% Ca and 4wt% Zn-0.2wt% Ca) show a fast corrosion rate due to several secondary elements in the alloy. As mentioned earlier, Ca exceeding the amount that can be completely dissolved in the Mg-Zn alloy contributes to the potential imbalance between the Mg matrix and Ca_2_Mg_6_Zn_3_. This can decrease the corrosion resistance of the alloy and cause local corrosion, such as pitting corrosion. Figure 10 shows the results of the micro-CT scan on the immersed tensile sample (0.1% Ca and 0.3% Ca). These results demonstrate that the decrease in mechanical properties after immersion is related to pitting corrosion. The 0.3% Ca alloy exhibited various pitting sizes of 20–240 μm, while the 0.1% Ca alloy had pit sizes less than 20 μm.

### Cytotoxicity test

**Fig. 11:**
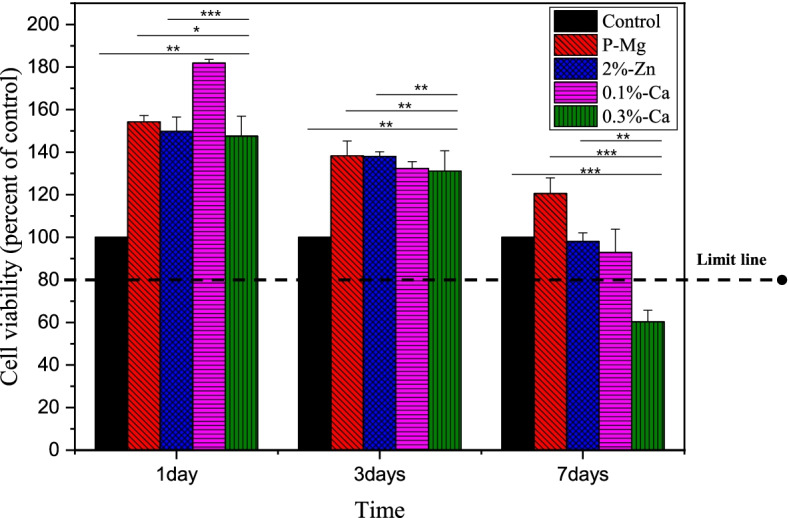
Cell viability of tested samples

Figure 11 shows the results of the cell viability test on the investigated samples. A basic media solution was used as a negative control. The control group was cultured in cell media for 24 h. Cell viability that falls below approximately 80% of the negative control is termed to have cytotoxicity. The results showed that the cell viability of all the samples (1 d) exceeded 100%. This implies that the extraction media does not affect cell viability. However, it could be considered that the extraction media can result in an increase in cell proliferation [29].

Cell viability decreased with time (1, 3, and 7 days) due to a complex set of factors, including pH and ionic activity. The degradation of Mg is related to the pH and ionic activity. Therefore, the low cell viability (< 80%) of the 0.3% Ca alloy could be associated with the high corrosion rate. Cells may find it difficult to survive in media with large amounts of Mg and Zn ions as well as alkaline conditions [30]. However, the 0.1% Ca alloy showed a cell viability of above 80% during the entire experimental period. This could be due to its better corrosion resistance.

## Discussion

In the Mg-2% Zn alloy system, the addition of Ca promotes the formation of the Ca_2_Mg_6_Zn_3_ phase, and as the amount of Ca added increases, the secondary phase along the grain boundary also increases. However, in the 0.3% Ca alloy, large amounts of Mg_2_Ca, in addition to Ca_2_Mg_6_Zn_3_, were detected through SEM analysis. Therefore, it is difficult for the 0.3% Ca to dissolve in the Mg matrix. In Fig. 1, the phases of Mg + liquid are stable in the equilibrium state at 416 ^°^C. Therefore, the secondary phase could have been a liquid phase, which then changed into a eutectic phase comprised of Mg_2_Ca and Ca_2_Mg_6_Zn_3_.

Microstructures after extrusion showed different secondary phases depending on the content of Ca. After the addition of 0.1% Ca, a fine and well dispersed secondary phase was observed, which did not form in the 2% Zn alloy. Cha et al. reported that this phenomenon is caused by dynamic precipitation during hot extrusion [7]. Moreover, coarse Mg_2_Ca was observed in the 0.3% Ca alloy. These results reveal that homogeneous distribution of the secondary phase is possible in the solid solution condition. In addition, decreased grain due to the addition of a small amount of Ca was observed through TEM.

In the immersion and electrochemical corrosion tests, the 2% Zn alloy showed the lowest corrosion rate, and the corrosion resistance gradually decreased as the amount of Ca added increased. Further, as found in the open circuit potential analysis, increasing the content of Ca results in a steady decrease of the Mg potential, due to the decreased solubility of Zn in the Mg matrix. This implies that Ca bridges a large potential gap between Ca_2_Mg_6_Zn_3_ and the Mg matrix, which may accelerate the anodic reactions of the Mg. Consequently, the potential differences between the secondary phases impact Mg corrosion behavior more than the volume fraction of the secondary phase.

The addition of 0.1% Ca had a surprisingly positive effect on the mechanical properties of the alloy. The small and well-dispersed Ca_2_Mg_6_Zn_3_ phase in the 0.1% Ca alloy (Fig. 3c) effectively retarded grain growth. It was also observed that the yield strength and elongation of the 0.1% Ca alloy increased by over 50% compared to those of the 2% Zn alloy. However, the decrease in mechanical properties, especially elongation, when 0.3% Ca was added, could have been caused by the secondary phase (located along the extrusion direction) acting as a crack trigger. It has been reported that a continuous secondary phase can improve mechanical strength but have negative effects on machinability [7, 31]. These results were in agreement with those of the corrosion test.

Moreover, the micro-CT scan demonstrated that the deterioration of mechanical properties after immersion is caused by pitting corrosion. Local stress concentration can occur easily in the 0.3% Ca alloy due to a potential imbalance between the Mg matrix and the secondary phases, contributing to the deterioration of the mechanical properties. Conversely, the 0.1% Ca alloy had a relatively slow corrosion rate and a uniform corrosion layer. This could cause the alloy to maintain its strength.

The 0.1% Ca alloy showed more stable cell viability than the 0.3% Ca alloy, which was related to corrosion and pH change. These results implied that managing the amount of Ca included in the Mg-Zn alloy is very important for improved mechanical and bio-corrosion properties for broad applications.

## Conclusion

This study investigated the influence of the addition of Ca on microstructural, mechanical, and corrosion properties of Mg-Zn alloys. A small addition of Ca results in the formation of small and well-dispersed Ca_2_Mg_6_Zn_3_ during hot extrusion. This gives rise to greater grain refinement and improved mechanical properties. The ultimate tensile strength and elongation of the 0.3% Ca alloy approached 350 MPa and 17%, respectively. However, the imbalance of the electrochemical potential between α-Mg and Ca_2_Mg_6_Zn_3_ due to the addition of Ca increases the corrosion rate. Further, the formation of Mg_2_Ca in the 0.3% Ca alloy during heat treatment accelerated local galvanic corrosion. With regard to the integrity of the alloys, the decrease in the mechanical properties of the 0.3% Ca alloy after immersion for 30 days was larger than that of the 0.1% Ca alloy. This is due to deeper pitting on the surface. The strength of the 0.1% Ca alloy was maintained even after immersion (YS:239.4 MPa and UTS:276.4 MPa). The 0.1% Ca alloy also demonstrated no cytotoxicity. These results implied that optimization of the Mg-Zn alloy system is possible through a small addition of Ca, which significantly improves mechanical properties while maintaining corrosion rate. This can open doors for a wide range of applications in orthopedic surgery.

## Data Availability

The datasets used and analyzed during the current study are available from the corresponding author upon reasonable request.

## Abbreviations

Mg: Magnesium
Ca: Calcium
Zn: Zinc
ICP: Inductively Coupled Plasma
SEM: Scanning Electron Microscopy
EDS: Energy Dispersive Spectroscopy
SADP: Selected Area Diffraction Pattern
TEM: Transmission Electron Microscopy
HBSS: Hank’s Balanced Salt Solution
OCP: Open Circuit Potential
DMEM: Dulbecco’s Modified Eagles Medium
UTS: Ultimate Strength; Micro-CT: Micro-Computed Tomography
