# Correction: Optimization of the clinically approved Mg-Zn alloy system through the addition of Ca

**DOI:** 10.1186/s40824-022-00333-y

**Published:** 2022-12-16

**Authors:** Hyung-Jin Roh, Jaeho Park, Sun-Hee Lee, Do-Hyang Kim, Gwang-Chul Lee, Hojeong Jeon, Minseong Chae, Kang-Sik Lee, Jeong-Yun Sun, Dong-Ho Lee, Hyung-Seop Han, Yu-Chan Kim

**Affiliations:** 1 Nanostructural Material Laboratory, Department of Advanced Materials, Yonsei University, Seoul, 03722 Republic of Korea; 2 Center for Biomaterials, Korea Institute of Science and Technology, Seoul, 02792 Republic of Korea; 3 Research and Development Center, U&I Corporation, Uijongbu, 480-050 Republic of Korea; 4 Department of Materials science and Engineering, Seoul National University, Seoul, 08826 Republic of Korea; 5 KU-KIST Graduate School of Converging Science and Technology, Korea University, Seoul, 02841 Republic of Korea; 6 Biomedical Engineering Research Center, Asan Institute for Life Sciences, Asan Medical Center, College of Medicine, University of Ulsan, Seoul, 05505 Republic of Korea; 7 Department of Orthopedic Surgery, Asan Medical Center, College of Medicine, University of Ulsan, Seoul, 05505 Republic of Korea

**Correction: Biomater Res 26, 41 (2022)**

**https://doi.org/10.1186/s40824-022-00283-5**

The original article [1] contained an error in a Funding attribution grant number which has since been amended.
